# Defects activated photoluminescence in two-dimensional semiconductors: interplay between bound, charged, and free excitons

**DOI:** 10.1038/srep02657

**Published:** 2013-09-13

**Authors:** Sefaattin Tongay, Joonki Suh, Can Ataca, Wen Fan, Alexander Luce, Jeong Seuk Kang, Jonathan Liu, Changhyun Ko, Rajamani Raghunathanan, Jian Zhou, Frank Ogletree, Jingbo Li, Jeffrey C. Grossman, Junqiao Wu

**Affiliations:** 1 Department of Materials Science and Engineering, University of California, Berkeley, California 94720, United States; 2 Institute of Semiconductors, Chinese Academy of Sciences, P.O. Box 912, Beijing 100083, People's Republic of China; 3 Department of Materials Science and Engineering, Massachusetts Institute of Technology, Cambridge, Massachusetts 02139, United States; 4 Materials Sciences Division, Lawrence Berkeley National Laboratory, Berkeley, California 94720, United States; 5 These authors contributed equally to this work.

## Abstract

Point defects in semiconductors can trap free charge carriers and localize excitons. The interaction between these defects and charge carriers becomes stronger at reduced dimensionalities, and is expected to greatly influence physical properties of the hosting material. We investigated effects of anion vacancies in monolayer transition metal dichalcogenides as two-dimensional (2D) semiconductors where the vacancies density is controlled by α-particle irradiation or thermal-annealing. We found a new, sub-bandgap emission peak as well as increase in overall photoluminescence intensity as a result of the vacancy generation. Interestingly, these effects are absent when measured in vacuum. We conclude that in opposite to conventional wisdom, optical quality at room temperature cannot be used as criteria to assess crystal quality of the 2D semiconductors. Our results not only shed light on defect and exciton physics of 2D semiconductors, but also offer a new route toward tailoring optical properties of 2D semiconductors by defect engineering.

In semiconductors, lattice point defects such as vacancies and interstitials can act as very efficient traps for electrons, holes and excitons, and strongly influence transport and optical properties of the host material. Excitons bound to defects, if recombine radiatively, lead to light emission at energies lower than the band-to-band optical transition energy. Such interactions become stronger in reduced dimensionalities due to tighter localization of the electron wavefunction. For example, in three dimensions (3D) within the hydrogenic defect model, shallow defects bind electrons at a ground-state binding energy equal to 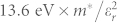, where m* is the effective mass and *ε_r_* is relative dielectric constant. This is increased to 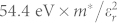 in two dimensions (2D) simply due to the dimensionality effect^1^. Similarly, the binding energy and recombination dynamics of Wannier and Frenkel excitons are expected to be drastically different going from 3D to 2D^2^,^3^,^4^. The escalated binding energy means stabilization of bound excitons and their emission features at higher temperatures, pointing to a potentially useful way to tailor optical properties of 2D semiconductors.

Although the understanding of point defects in conventional 3D semiconductors is well established and the defects database is relatively complete, physics and behaviour of point defects in 2D semiconductors, such as the newly emerging monolayer semiconducting transition metal dichalcogenides (TMDs), have remained an unexplored field. In this work we report the effects of anion vacancies, the dominant point defect species, on photoluminescence (PL) and Raman spectra of monolayer TMDs. Since these materials become direct-bandgap semiconductors with relatively high PL intensity in the monolayer limit^5^, the defect effects can be easily monitored optically. We find that irradiation with MeV α particles or thermal annealing at sub-decomposition temperatures introduce anion vacancies in monolayer MoS_2_, MoSe_2_, and WSe_2_, where the vacancy density can be controlled by the irradiation dose or annealing time. These defects introduce a new emission peak at ~0.15 to 0.25 eV below the free-exciton PL peak, and its intensity is enhanced as the defect density is increased. Moreover, the overall PL intensity also increases at higher defect densities, and as such, the defective material becomes *more* luminescent compared to pristine monolayers. Surprisingly, these effects are absent when measured in vacuum, suggesting that the interaction between ambient gas molecules and the defect sites play a significant role in the process. Our density functional theory (DFT) calculations show that these anion vacancies create energy levels approximately 0.2 eV below the band edge, and gas molecules can be physically adsorbed at the defect sites with relatively large charge transfer, which electron-depletes the material.

## Results

**Figure 1:**
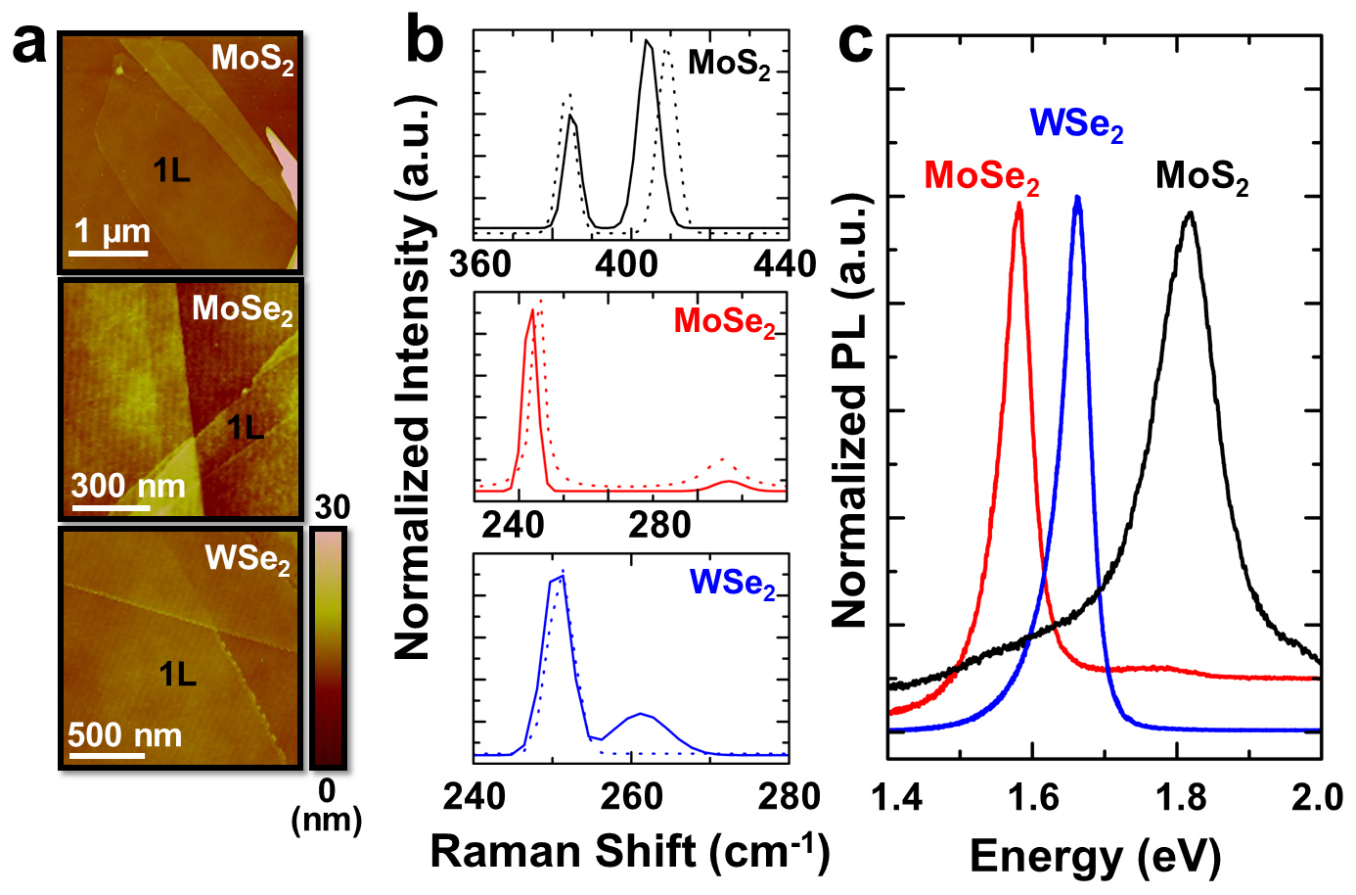
(a). AFM images taken on monolayer MoS_2_, MoSe_2_, and WSe_2_. (b). Raman spectrum measured on monolayer MoS_2_, MoSe_2_, and WSe_2_, where the solid and dashed curves correspond to monolayers and few-layers, respectively. (c). Room-temperature normalized PL for monolayer MoS_2_, MoSe_2_, and WSe_2_.

**Figure 2:**
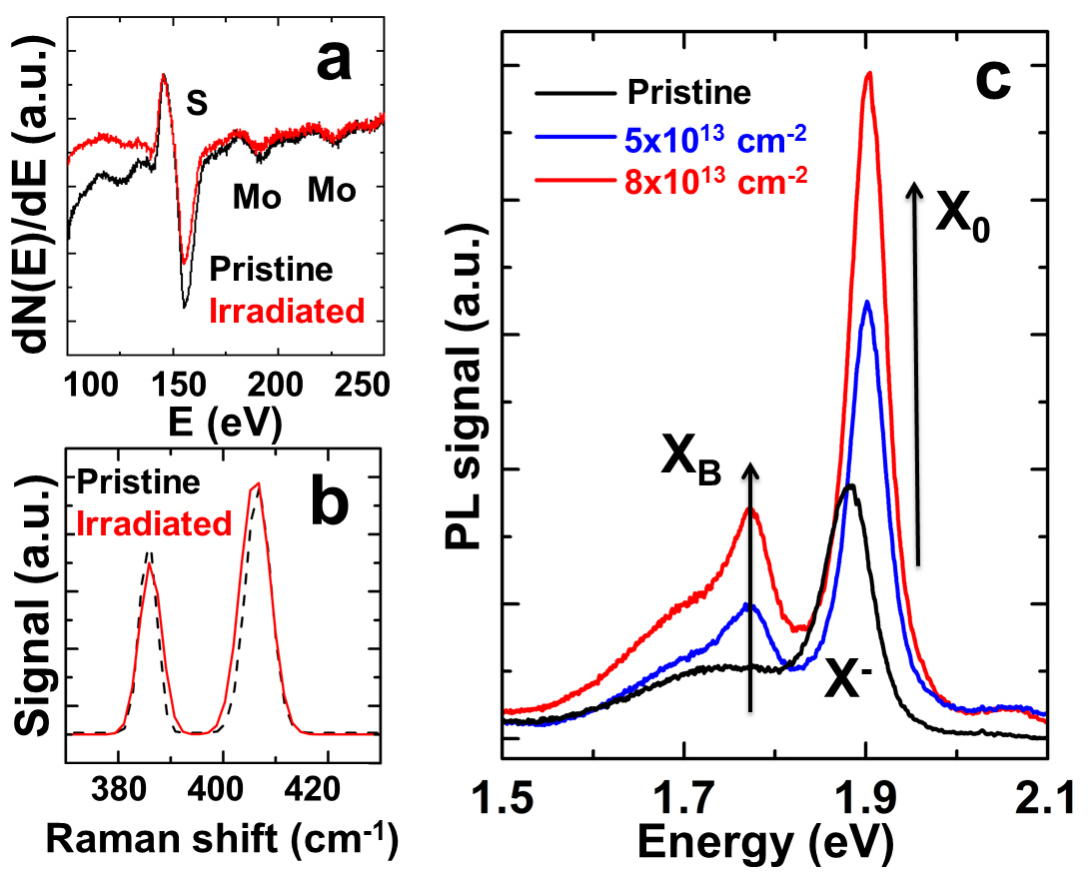
(a). Nano-Auger spectrum taken on a monolayer MoS_2_ before and after irradiation with α particles at a dose of 8 × 10^13^ cm^−2^. (b). Raman spectrum of the same. (c). PL spectrum for pristine and irradiated monolayer MoS_2_ at the shown irradiation doses. The PL was taken at 77 K in N_2_ (50 Torr) environment with a constant laser excitation power. The irradiation-caused enhancement in bound exciton (X_B_) and free exciton (X_0_) emission intensity is indicated.

Monolayer MoS_2_, MoSe_2_, and WSe_2_ flakes were mechanically exfoliated from bulk crystals onto 90 nm SiO_2_ where a relatively high contrast can be observed at the flakes^6^. The monolayers were identified using atomic force microscopy (AFM), Raman, and PL measurements. AFM measurements on monolayers yield a height of ~0.7 nm corresponding to the thickness of a single unit cell (Figure 1a). From the bulk to monolayers, the out-of-plane Raman mode (A_1g_) softens and the in-plane mode (E_2g_) stiffens for MoS_2_^7^ and MoSe_2_^8^,^9^, whereas for WSe_2_^10^, the degenerate A_1g_ and E_2g_ modes in the bulk split by 12 cm^−1^ in the monolayer as a result of broken degeneracy (Figure 1b). The PL signal is greatly enhanced by orders of magnitude from the bulk to monolayer due to the indirect to direct bandgap transition, consistent with previous reports^5^,^8^,^11^. At room temperature, monolayer MoS_2_, MoSe_2_, and WSe_2_ show a strong PL peak at 1.84 eV, 1.56 eV and 1.65 eV, respectively (Figure 1c). The exfoliated monolayers were irradiated with 3 MeV α particles at controlled doses to create different densities of point defects, and the samples were cooled down to the lowest attainable temperature in our system (77 K) for PL and Raman measurements. Before and after the irradiation, the sulfur to molybdenum (S/Mo) atomic ratio was monitored using nano-Auger electron spectroscopy (nano-AES). We find that the S/Mo ratio decreases slightly (Figure 2a), implying that the irradiation induces S vacancies in the 2D crystal, consistent with earlier results^12^. After the irradiation, the full-width-at-half maximum of the Raman peaks slightly broadens (Figure 2b) due to relaxation of the Raman selection rule at the defects.

At 77 K, the as-exfoliated MoS_2_ monolayers display a strong PL peak at 1.90 eV corresponding to the direct bandgap at the K symmetry point. Upon the α particle irradiation at different doses, the 77-K PL spectrum changes significantly as shown in Figure 2c and these changes are summarized as follows: (1) a new PL peak appears at 1.78 eV and the integrated intensity of this peak increases with the irradiation dose; and (2) the integrated intensity of the main PL line at 1.90 eV increases by ~3 times while the PL peak position shifts to higher energy by ~20 meV. The Stopping and Range of Ions in Matter (SRIM) calculations estimate that 7.5 × 10^−3^ vacancy/cm × ion is generated on MoS_2_ upon the α particle irradiation, and this corresponds to 6 × 10^11^ cm^−2^ defect density, or approximately one defect per 100 unit cells, for 8 × 10^13^ cm^−2^ irradiation dose. We note that here the PL was all measured in the presence of N_2_ gas (~50 Torr), and the effect of N_2_ will be discussed later. The small blueshift and enhancement in the main PL intensity bear much resemblance with the previously reported transition from charged exciton (X^−^ or eeh) to neutral free exciton (X_0_ or eh) in 2D systems, such as 2D electron gas (2DEG) heterostructures^13^ and more recently on monolayer TMDs^2^,^3^,^4^. In these cases, the charged to neutral exciton transition is associated with charge depletion^2^,^3^,^4^ or charge localization^13^ which stabilizes (destabilizes) neutral (charged) excitons. In accord with these studies, we attribute the observed blueshift to irradiation-induced defect sites interacting with N_2_ molecules, resulting in depletion and localization of charge carriers in the monolayer TMDs. Such effects will be discussed more in detail later.

**Figure 3:**
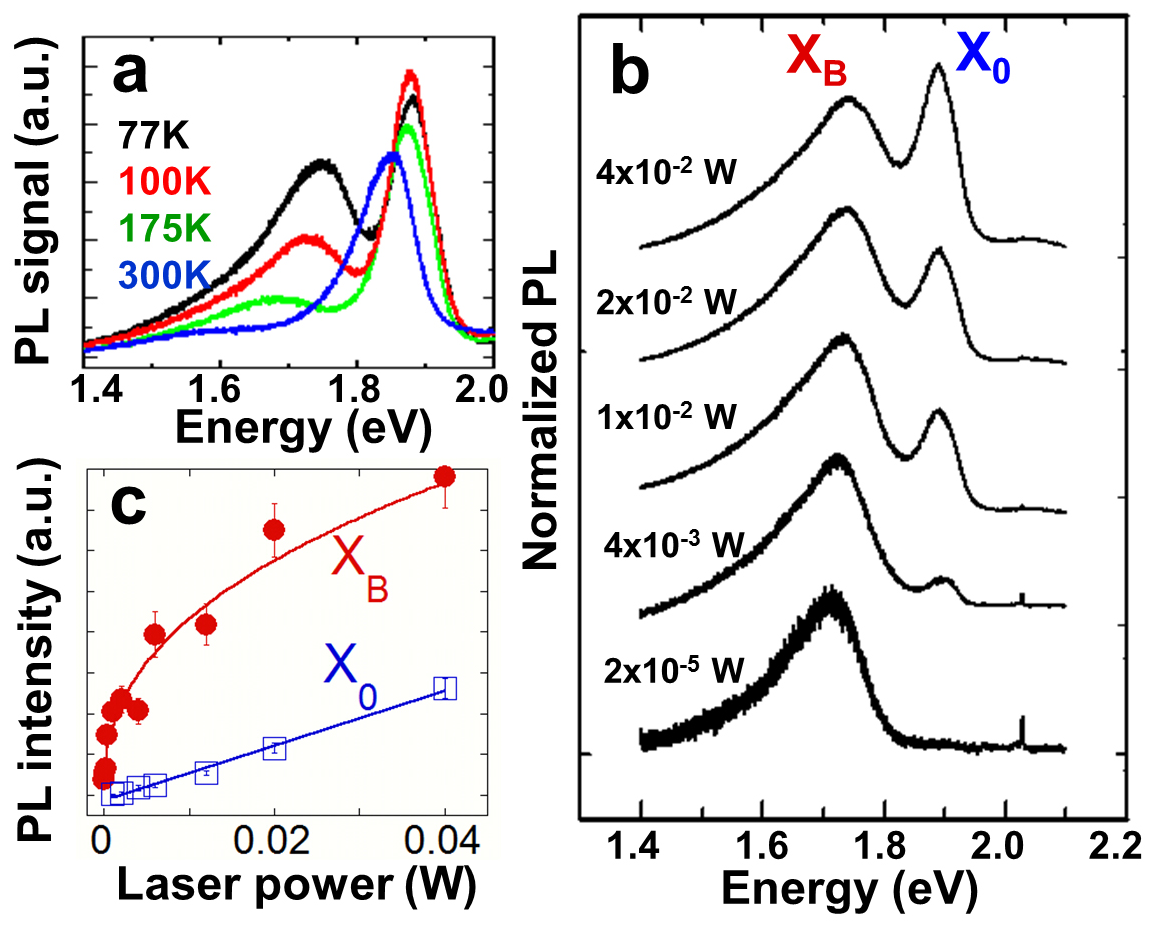
(a). PL spectrum measured over the temperature range from 77 K to 300 K of a monolayer MoS_2_ after being annealed to 500°C. (b). The PL at 77 K with different excitation laser power. Both a and b were taken in the presence of N_2_ gas (50 Torr). (c). Integrated PL intensity of bound exciton (X_B_) and free exciton (X_0_) as a function of excitation laser power.

Next, we focus on the new peak at 1.78 eV (Figure 1c). Since this PL peak appears after the irradiation and its intensity increases at higher doses, we attribute it to radiative recombination of bound excitons (X_B_), i.e. neutral excitons (X^0^) bound to defects. To probe this more, we also introduced the defects by thermal annealing in vacuum. Here, monolayers were annealed ~100°C below their thermal decomposition temperature (~600°C), and this process is known to create S vacancies in MoS_2_^14^. After the annealing, the PL spectrum also displays a defect-induced bound exciton peak at ~1.78 eV (Figure 3a), except that the peak appears relatively stronger and broader than in the irradiated samples, possibly due to a higher density of point defects and defect clusters created by annealing. It is expected that vacancies generation by particle irradiation is a series of highly non-equilibrium, random and isolated events, while thermal annealing is much slower and may facilitate formation of vacancy clusters with different configurations. These defect complexes with different clustering configurations may have different exciton binding energies, thus broadening the observed defect PL peak.

Further confirmation on the defect origin of the 1.78 eV peak comes from its excitation power dependence (Figure 3c). Since the X_B_ is associated with excitons bound to defects, the PL intensity of X_B_ is expected to saturate at high excitation power intensities when these defects are fully populated with excitons^15^. Consistent with this, the intensity of X_B_ exhibits a sub-linear laser power dependence with a tendency to saturate at high excitation powers, whereas the free exciton intensity (X_0_) scales linearly without any sign of saturation (Figure 3b–c). As a result, the overall PL spectrum is mostly dominated by the defect peak, X_B_, at low excitation intensities, and the X_0_ line becomes observable only at high excitation intensities (Figure 3b).

**Figure 4:**
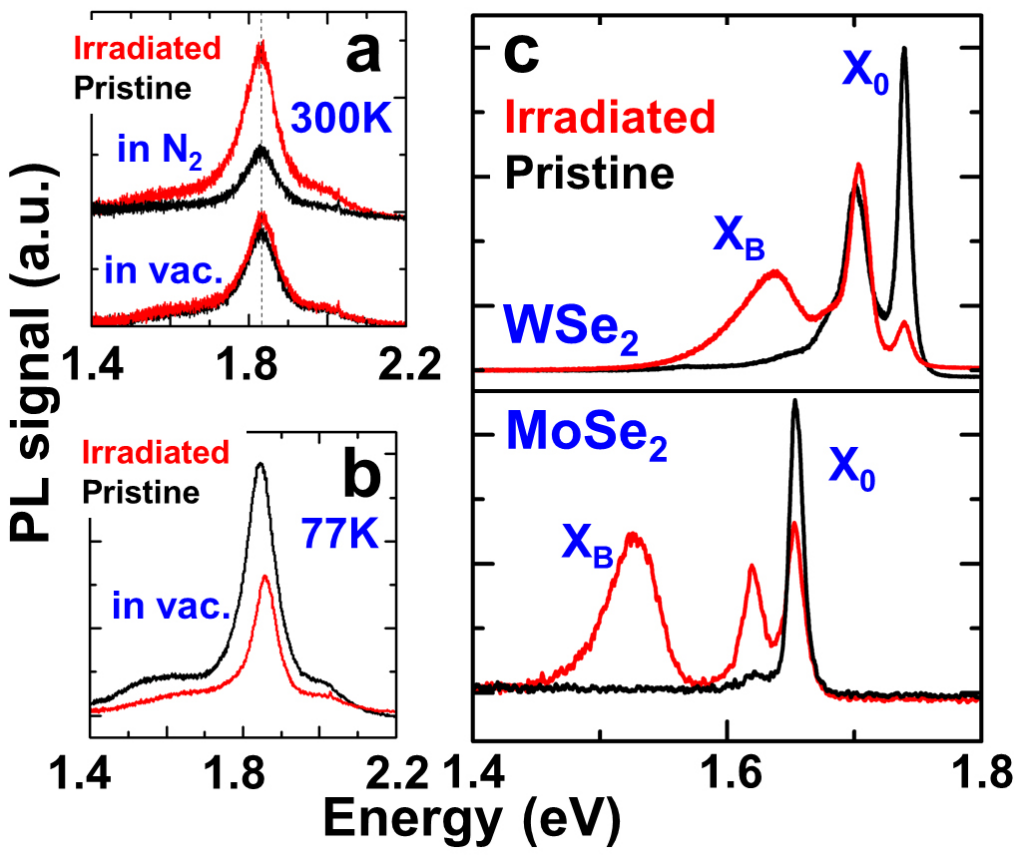
(a). PL spectrum of a monolayer MoS_2_ at 300 K in the presence of N_2_ or in vacuum before and after irradiation (dose ~ 8 × 10^13^ cm^−2^). (b). The same taken at 77 K in vacuum. (c). PL spectrum taken at 77 K in N_2_ on WSe_2_ and MoSe_2_ monolayers before and after the irradiation.

Next, we consider the effects of temperature and interaction with ambient gas molecules on the PL spectrum of defective monolayers. First, we note that the X_B_ peak is prominent at low temperatures or low excitation powers (Figure 3a–b). It becomes weaker as the temperature is increased and completely disappears above 250 K. At room temperature, both pristine and defective monolayers exhibit high optical quality with a strong, single PL peak associated with the band-to-band optical transition at the K point. Therefore, we conclude that in opposite to conventional wisdom believed in this new field of 2D semiconductors^16^,^17^, optical quality at room temperature (PL intensity and sharpness) cannot be used as criteria to assess the crystal quality of the monolayers. Indeed, the defective monolayers yield even stronger PL intensity at room temperature (Figure 4a). However, the PL spectrum at 77 K immediately tells the difference between the pristine and defective monolayers (Figure 2c).

The PL spectra discussed so far were all recorded in the presence of N_2_ gas, regardless of the measurement temperature (300 K or 77 K). Interestingly, when measured in vacuum, the aforementioned defect peak (X_B_) in the PL spectrum disappears at both room and low temperatures (Figure 4a–b). We also note that the occurring of the defect PL peak was instantaneously reversible when the chamber is purged with or pumped out of N_2_. This implies that the interaction between the defect sites and the N_2_ gas molecules is weak (physi-sorbed), but dictates the optical emission of the material. The above results are discussed in detail in conjunction with first-principles calculations shown below.

## Discussion

**Figure 5:**
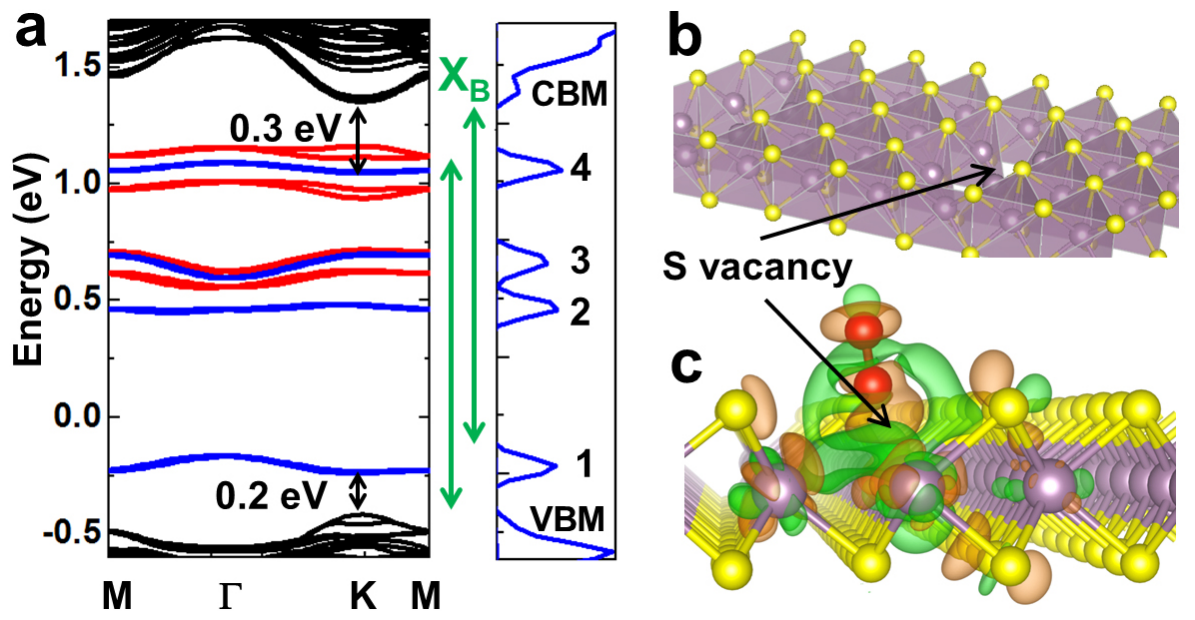
(a). Left panel: Calculated band structure of monolayer MoS_2_ in the presence of di-S vacancies. Red levels within the bandgap are the levels appearing when the S vacancies are introduced. Blue levels appear when the N_2_ molecule interacts with the S vacancies. Right panel: Total density of states of the monolayer MoS_2_ with S vacancies in the presence of N_2_. Here the modelled vacancies density is 7 × 10^13^/cm^2^. (b). Monolayer MoS_2_ in the polyhedral representation to illustrate the di-S vacancy. (c). Charge density plots (iso-surface value = 10^−3^ e/Å^3^) of monolayer MoS_2_ with a di-S vacancy interacting with a N_2_ molecule (red). Orange denotes charge accumulation and green charge depletion.

To understand the physical origin of the defect-induced PL peak (X_B_) and the intensity enhancement in the main PL (X_0_), we calculated the band structure and the density of states (DOS) of defective monolayer MoS_2_. Since the α particle irradiation^12^,^18^ and thermal annealing may result in various types of sulfur vacancies in MoS_2_, the monolayer MoS_2_ was modelled in the presence of sulfur vacancies in different arrangements (Supplementary Information Fig. S1). However, our calculations show that di-sulfur vacancies are most relevant to the experiments presented here, so our discussions will be limited to the di-S vacancies. In Figure 5a, we show the band structure of a monolayer MoS_2_ in the presence of di-S vacancies with and without the N_2_ gas molecules around. We first note that once the di-S vacancies are created, new states appear within the bandgap. Specifically, states with energies 0.2 ~ 0.3 eV near the conduction/valence band edge (Figure 5a red) are of particular interest, as they are close to the energy difference between X_B_ and X_0_ (0.12 eV). However, since the X_B_ peak is observable only in the presence of N_2_ gas molecules, defect-induced levels themselves are not enough to explain our results. Next, we take into account the interaction with N_2_ molecules at the defect sites (Figure 5a blue) and our findings are summarized as follows;

1. In the absence of S vacancies, the interaction between the pristine monolayer MoS_2_ and N_2_ is negligible. N_2_ molecules electronically interact with the MoS_2_ only at the defect sites. The calculated binding energy of such interaction varies from 90 meV to 150 meV, depending on the type of the S vacancy, implying that the N_2_ molecules are physi-sorbed (as opposed to chemi-sorbed) at the defect sites. As a result, the effect of N_2_ is reversible when the chamber is purged with or pumped out of N_2_.
2. The interaction of a N_2_ molecule with the di-S vacancy on a 4 × 4 unit cell results in ~0.2e charge transfer from MoS_2_ to the N_2_ molecule. The N_2_ molecules deplete the material using the defect sites as channels, and the total free carrier density is much reduced. Consequently, screening on the excitons are lifted, hence the free neutral (X_0_) and bound (X_B_) neutral excitons are stabilized, while the negatively charged excitons (X^−^) vanish due to the lack of equilibrium free electrons in the system to act as the second electron in the X^−^ 13. As a result, the intrinsic free exciton line (X_0_) is enhanced, and the X_B_ peak is observed.
3. The discrete energy levels that are introduced by the S vacancies (Figure 5 red) are renormalized by the N_2_ interacting with the defect sites (Figure 5a blue). Afterwards, two levels appear at ~0.2 eV above the valence band maximum (VBM) (labelled #1) and ~0.3 eV below the conduction band minimum (CBM) (labelled #4). Optical transitions from CBM to #1 and #4 to VMB yield a PL peak that is 0.2 eV and 0.3 eV lower than the X_0_ peak. Based on these comparisons, we attribute the bound exciton peak X_B_ to either or both these transitions as shown in Figure 5a.

The onset of the X_B_ emission is not specific to N_2_ gas, but to other gas species that are known to deplete the monolayer TMDs, such as O_2_^19^. Our DFT calculations predict that O_2_ molecules are physi-sorbed at the MoS_2_ anion vacancy sites with a binding energy ~300 meV and withdraws 0.6 electrons per 4 × 4 supercell from the monolayers; therefore, the interaction with O_2_ is stronger than N_2_ at the defect sites. In this case, interaction with the O_2_ molecules introduces various discrete energy levels within the bandgap, some of which are ~150–300 meV below the band gap. Consistent with these DFT predictions, our experiments with O_2_ indeed show a X_B_ peak located at 1.75 eV, and possibly more peaks at the low-energy shoulder (Supplementary Information Fig. S2).

Point defects exist ubiquitously to different extents even in pristine 2D semiconductors. The observed effects of point defects are universal in other 2D semiconductors as well. Similar to MoS_2_, after irradiating MoSe_2_ and WSe_2_ monolayers with α particles, a broad bound exciton peak appears below the bandgap transition, as shown in Figure 4c. The bound exciton peak is located at 1.52 eV and 1.63 eV for MoSe_2_ and WSe_2_, respectively, which is about 0.10 eV below their bandgap. These results indicate that the reported interaction of gas molecules with defect sites and the resultant influence on the optical property are a general effect applicable to a variety of monolayer TMDs, as well as different environmental molecules (O_2_ and N_2_).

In conclusion, anion vacancies as point defects drastically modify the optical properties of monolayer TMDs in such a way that: (1) the overall integrated PL intensity is enhanced, and (2) a new, defect-related peak is observed below the bandgap. These effects are prominent at low temperatures and in gas environments (such as N_2_). We show that the new peak originates from bound excitons that are formed by localizing excitons at the defect sites. The overall enhancement in the PL intensity is attributed to an electronic effect of the defects: the gas molecules drain free electrons from the material via these defect sites, causing a transition of exciton population from charged to neutral (both free and bound) excitons. Our results not only shed light on defect and exciton physics of 2D semiconductors in general, but also offer a new route toward tailoring their physical properties by defect engineering. The latter includes, for example, doping or compensating 2D semiconductors with irradiation, potentially creating 2D multi-bandgap semiconductors for wide-spectrum response akin to those enabled by defect-engineered 3D semiconductors^20^, fabricating multi-coloured light emission devices by controlling defects inside a single 2D semiconductor, and photometric-spectrally resolved optical sensors for sensing gas molecules and/or radiative environment.

## Methods

### Sample preparation, micro-PL/Raman, and AFM measurements

Monolayer MoX_2_ flakes were exfoliated from bulk crystals (2Dsemiconductors and SPI) onto Si wafer (MTI corporation, resistivity 0.001 ~ 0.1 Ωcm) with 90 nm thermal oxide. Measurements were performed using a Renishaw micro-PL/Raman system. The laser beam (wavelength 488 nm) was focused onto the sample (spot diameter of ~1–2 μm) using excitation power up to 5–10 μW unless stated otherwise in the manuscript. The PL/Raman measurements were performed in a home-made vacuum chamber pumped down to ~10^−4^ Torr using a turbo-molecular pump. High purity (99.9995%) N_2_ gas was introduced into the chamber regulated by flow meters and the pressure was measured by a vacuum gauge. During the measurements, extensive precautions were taken to eliminate the contamination, and the base pressure of the residual gas remained below 3 × 10^−5^ Torr. AFM measurements were performed using Veeco Multimode Atomic Force Microscope in the tapping mode. All results were reproduced on more than ten samples.

### α particle irradiation and thermal annealing

The exfoliated monolayer samples were irradiated employing a high energy (3.04 MeV) He^2+^ beam with current around 35 nA generated by a Pelletron tandem accelerator. The ion beam was defocused to an area of 32 mm^2^ maintaining a homogeneous ion fluence over the entire flake. The accumulated dose was monitored by Faraday cups inside the irradiation chamber. For the annealing, the samples were heated to 500°C at 30°C/min rate and the temperature was hold at 500 ± 0.5°C for 30 minutes. The annealing was performed in a 2-inch quartz tube in vacuum (30 mTorr base pressure). Prior to the annealing, the quartz tube was cleaned at 1000°C in H_2_ gas (2 Torr) overnight.

### Density functional theory calculations

Our calculations were based on first-principles density functional theory (DFT) using projector augmented wave potentials^21^. The exchange correlation potential has been represented by the generalized gradient approximation characterized by Perdew-Burke-Ernzerhof^22^ including van der Waals corrections^23^ both for spin-polarized and spin-unpolarized cases. Effects of spin-orbit coupling and non-collinear magnetism were taken into account in the spin-polarized calculations. The super-cell size, kinetic energy cut-off, and Brillouin zone sampling of the calculations were determined after extensive convergence analyses. A large spacing of ~15 Å between the 2D single layers was used to prevent interlayer interactions. A plane-wave basis set with kinetic energy cut-off of 300 eV was used. In the self-consistent field potential and total energy calculations, the Brillouin zone was sampled by special k-points. The numbers of these k-points were (25 × 25 × 1) for the primitive 1H-MoS_2_ and were scaled according to the size of the super cells. All atomic positions and lattice constants were optimized using the conjugate gradient method, where the total energy and atomic forces were minimized. The convergence for energy were chosen to be 10^−6^ eV between two consecutive steps, and the maximum Hellmann-Feynman forces acting on each atom was less than 0.01 eV/Å upon ionic relaxation. The pressure in the unit cell was kept below 5 kbar. Numerical calculations were performed by using the VASP software^24^.

## Author Contributions

S.T. and J.W. conceived the project. S.T., J.S., F.W., A.L., J.S.K., J.L. and J.Z. performed the measurements. C.A., R.R. and J.C.G. performed the density functional theory calculations. C.K. and F.O. performed nano-Auger measurements. S.T. and J.W. wrote the manuscript. All authors have read the manuscript.

## Supplementary Material

Supplementary Information Supplementary Information
