# Supplementary material for: Defects activated photoluminescence in two-dimensional semiconductors: interplay between bound, charged, and free excitons
Source: Sci Rep. 2013 Sep 13;3:2657. doi: 10.1038/srep02657 (PMC3772378; doi:10.1038/srep02657)
Supplement: Supplementary Information [file srep02657-s1.pdf]

Supplementary Information for

Defects activated photoluminescence in two-dimensional semiconductors: interplay between bound, charged, and free excitons

Sefaattin Tongay [1, 2 +], Joonki Suh [1, 2 +], Can Ataca [3], Wen Fan [1], Alexander Luce [1, 4], Jeong Seuk Kang [1], Jonathan Liu [1], Changhyun Ko [1], Rajamani Raghunathanan[3], Jian Zhou [1], Frank Ogletree[4], Jingbo Li [2], Jeffrey C. Grossman [3] and Junqiao Wu[1, 2, 4 *]

[1] Department of Materials Science and Engineering, University of California, Berkeley, California 94720, United States

[2] Institute of Semiconductors, Chinese Academy of Sciences, P.O. Box 912, Beijing 100083, People's Republic of China

[3] Department of Materials Science and Engineering, Massachusetts Institute of Technology, Cambridge, Massachusetts 02139, United States

[4] Materials Sciences Division, Lawrence Berkeley National Laboratory, Berkeley, California 94720, United States

I. Density Functional Theory (DFT) Calculations: Effects of various S-vacancy configurations.

In our measurements the sensitivity to the N_2 gas molecules are reversible with a time response limited to purging / pumping speed. Therefore, the binding between the monolayer Transition Metal Dichalcogenides (TMDs) and N_2 gas has to be physi-sorption in origin. We have calculated the electronic band structure of monolayer MoS_2 in vacuum and N_2 rich environment before / after introducing the chalcogen vacancies. The binding energies of the N_2 gas molecules to monolayers were given by $E_b=E_{MoS2+ad}-E_{MoS2}-E_{ad}$ where $E_{MoS2+ad}$ is the total energy of relaxed structure of single layer MoS_2 and a N_2 molecule, E_{MoS2} and E_{ad} are the total energies of single layer MoS_2 and N_2 molecule, respectively. Our calculations on monolayer MoS_2 were performed on 4×4 supercell including the spin-orbit interaction and magnetic

coupling interactions. Even though N_2 molecule is physi-sorbed on MoS_2 by E_b~70 meV, the molecular levels of N_2 are ~10-15 eV lower than Fermi energy of MoS_2 and therefore binding N_2 on monolayer MoS_2 has no influence on its photoluminescence peak position, lineshape, and magnitude

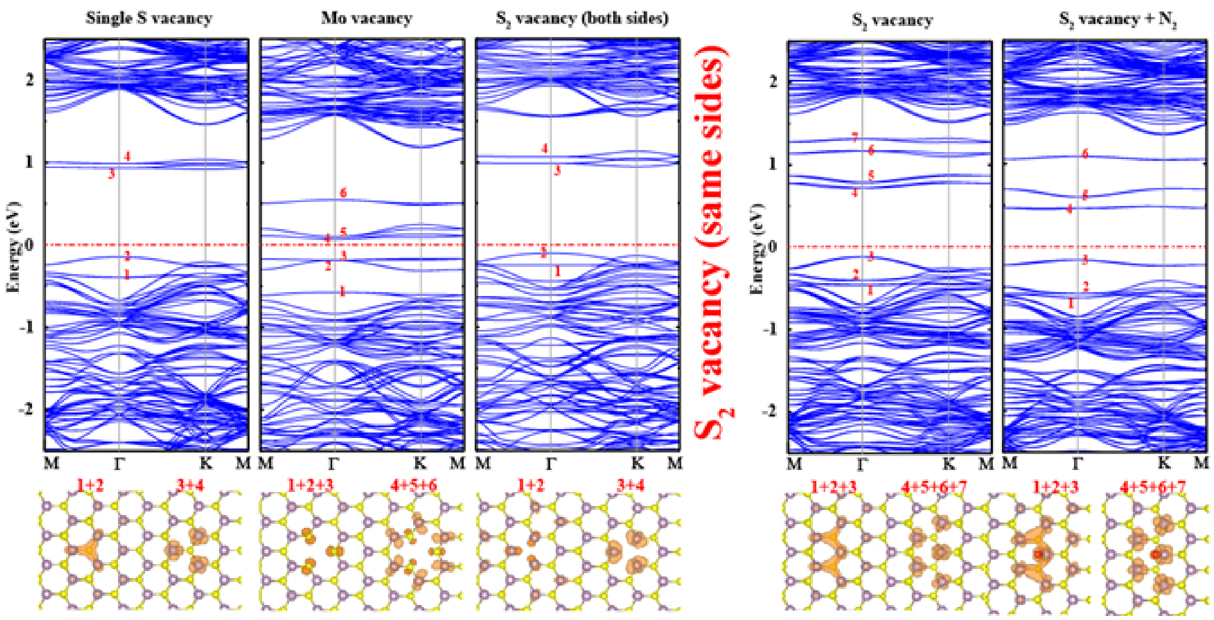

Fig.S1 (Top panels) Band structure of monolayer MoS_2 in the presence of various types of vacancies. (Bottom panels) The charge density plots for each localized bands. Each charge density graph is made for localized bands within the bandgap. Isosurface value is taken as $7x10^{-5}$ e/A^3

α particle radiation onto the surface of single layer MoS_2 and thermal annealing below decomposition temperature [1] can create vacancy defects on single layer MoS_2 and probably new physi-sorption sites for the N_2 molecules. It is known that vacancy defects in two-dimensional graphene [2-6], graphene nanoribbons[7,8], two-dimensional graphane [9] and graphane nanoribbons [10] give rise to crucial changes in the electronic and magnetic structure. We investigate four different types of vacancy defects that are formed on single-layer MoS_2, namely Mo and S single vacancies, two different geometric configurations of S_2 double-vacancies. These are two neighboring S vacancies on the same side of MoS_2 (same-side) and two neighboring S vacancies on different sides of MoS_2, but on top of each other (both-sides). Larger vacancy defects including MoS double-vacancies and MoS_2 triple-vacancies are not included in

this study as larger defects are less probable to create, and generate attractive regions where molecules can get trapped and even dissociate into constituting atoms [11,12]. All structures were optimized upon the creation of a particular type of vacancy. Vacancy energies, E_V, were calculated by subtracting the total energy of the perfect structure (without any vacancy) from the sum of the total energy of a structure having a particular type of vacancy and the total energy(ies) of missing atoms in the vacancy defect. The vacancy formation energies are 6.04, 14.13, 12.00 and 11.96 eV for S, Mo, S_2 (same-side) and S_2 (both-sides), respectively. It is energetically favorable by 80 meV to have S_2 double-vacancy in the same side of MoS_2 than having 2 separate non-interacting S vacancy defects.

On Fig. S2, we show the energy band structure of MoS_2 monolayers with different types of S and Mo vacancies. After the vacancy is created, charges become localized around the vacancy regions as shown in the charge density plots and localized states are created within the band gap. These additional states introduce an intermediate level(s) for free excitons to relax to and further relaxation (radiative recombination) from these levels to VBM results in a new PL peak that is below the band edge luminescence. We first note that mono-chalcogen (S), di-chalcogen (S_2) (on both sides of MoS_2), and Mo vacancies introduce energy levels that is at least 0.4 eV below the band edge. However, these bands lay 0.2 eV below the CBM for MoS_2 with di-chalcogen vacancies (same side). We believe that the new PL peak appearing 0.15 eV below the free exciton PL at 1.9 eV is associated with the same-side di-chalcogen (S_2) vacancies whereas other types of vacancies only cause overall broadening in the bound exciton peak.

Even though the same-side di-chalcogen vacancy provides an explanation for the peak position of the bound exciton peak, the bound exciton peak is only observable under N_2 gas conditions. For each vacancy defect, we carried out geometry relaxations for at least five different initial N_2 absorbed geometries to find the energetically most favorable absorption site. For single-vacancy defects (Mo and S), the only energetically favorable absorption site is ~ 0.2 nm above vacancy defect region where the N_2 molecule is parallel to MoS_2 surface. The calculated binding energies of N_2 molecule are 115 and 81 meV for S and Mo single-vacancy defects, respectively. Calculated energy band structures are the same as the ones without N_2 molecules around the Fermi energy (± 5 eV), since there is no significant interaction and charge transfer. Localized N_2 molecule states are ~10-15 eV lower than Fermi Energy. In the case of S_2

(both-sides) double-vacancy defects, there are two energetically probable absorption sites. One is similar with single-vacancy defects, where N_2 molecule is 0.2 nm away from the surface with a binding energy of 118 meV and has no effect on the electronic structure. In the other absorption geometry, N_2 takes the place of vacant S_2 sites and makes a strong bond with surrounding Mo atoms. Very high binding energy of 2.28 eV makes this absorption cite least probable for desorption of N_2. As S layers in MoS_2 act as charge accumulation regions, the N_2 molecule is 1.57 electrons negatively charged. In case of S_2 (same-side) double-vacancy defects, absorption geometry shown in Fig. 4 c and d is the energetically most favorable site with a binding energy of 143 meV. When N_2 is physi-sorbed in this vacancy defect, 0.24 electrons accumulate on the N_2 molecule. The energy band structure shows minor changes in the localized states. Due to its low binding energy and comparable energies of localized states with first-principles *ab-initio* calculations and experiments, we conclude that clustered S vacancy defects on the same side of single-layer MoS_2, can provide physi-sorption sites for N_2 molecules and induce localized states which create new radiative channels.

II. Effects of interaction with O_2 molecules at the defect sites

Our DFT calculations show that the interaction with O_2 molecules at different types of vacancy types results in binding energies typically ranging from 300 meV to 8eV. In most of the cases, binding O_2 molecule to the defective MoS_2 requires large energy to overcome the energy barrier (2 ~ 3eV). We find that O_2 molecules only get physi-sorbed at the di-sulfur vacancy site with ~300 meV binding energy. Once the O_2 is absorbed at the surface, additional states appear within the bandgap and some of levels are within ~200 meV vicinity to conduction and valance bands (Figure S2). Similar to the case of N_2, transitions from these levels to conduction and valance bands are expected to yield X_B emission line(s).

Consistent with the DFT predictions, the PL spectrum taken from irradiated MoS_2 in O_2 gas display a X_B peak 1.75 eV, and possibly more peaks at lower energies but cannot be individually resolved at 77K. Even though the overall PL looks similar to the N_2 case, the X_B line is weaker and the X_0 line peak position is slightly different. These results show that the reported interaction of gas molecules with defect sites is a general effect applicable to a variety of monolayer TMDs (such as MoS_2, $MoSe_2$ and WSe_2), as well as different molecules (O_2 and N_2).

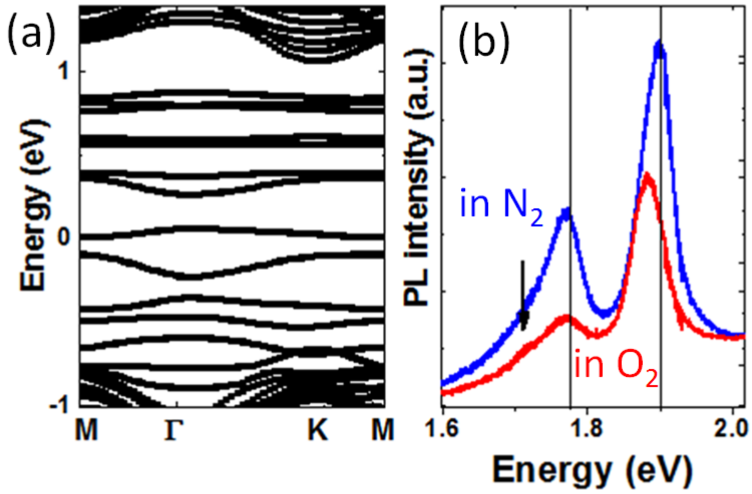

Fig.S2 (a) Band structure of monolayer MoS_2 in the presence of di-sulfur vacancies interacting with O_2 molecules. (b) Comparison between PL spectra taken on irradiated MoS_2 monolayer in the presence of O_2 (red) and N_2 (blue) gases. The O_2 effect is only observable when the O_2 partial pressure is above 100mT.

References

[1] Hao Qiu et al. Electrical characterization of back-gated bi-layer MoS2 field-effect transistors and the effect of ambient on their performances, Appl. Phys. Lett. 100, 123104 (2012)

[2] Esquinazi, P.; Spemann, D.; Hohne, R.; Setzer, A.; Han, K.; Butz, T. Phys. Rev. Lett. 91, 227201 (2003).

[3] Hashimoto, A.; Suenaga, K.; Gloter, A.; Urita, K.; Iijima, S. Nature, 430, 870 (2004).

[4] Yazyev,O.V.;Helm,L.Phys.Rev.B,75, 125408 (2007).

[5] Vozmediano, M.; Lopez-Sancho, M.; Stauber, T.; Guinea, F. Phys. Rev. B, 72, 155121 (2005).

[6] Brey, L.; Fertig, H. A.; Das Sarma, S. Phys. Rev. Lett. , 99, 116802 (2007).

[7] Palacios,J.J.;Fernandez-Rossier,J.; Brey,L. Phys. Rev. B, 77,195428 (2008).

[8] Topsakal, M.; Akturk, E.; Sevincli, H.; Ciraci, S. Phys. Rev. B, 78, 235435 (2008).

[9] Sahin, H.; Ataca, C.; Ciraci, S. Appl. Phys. Lett., 95, 222510 (2009).

[10] Sahin,H.; Ataca,C.; Ciraci, S. Phys. Rev. B, 81, 205417 (2010).

[11] Ataca, C.; Ciraci S. J. Phys. Chem C,115, 13303 (2011).

[12] Ataca, C.; Ciraci S. Phys. Rev. B 2012, 85, 195410 (2012).
